# Society, Parturition and Posterity

**Published:** 1902-02

**Authors:** Ralph Methven Thomson

**Affiliations:** Savannah, Ga.


### SOCIETY, PARTURITION AND POSTERITY.

By RALPH METHVEN THOMSON, M.D.,

SAVANNAH, GA.

Considered from a medical standpoint, do the "signs of the times" point toward the amelioration and perpetuity of the highly educated, the well-to-do and fortune-favored members of the human family; or, on the other hand, to their disintegration and ultimate extermination? Notwithstanding our boasted civilization and progress, is the nefarious practice so often indulged in by modern women of affluent circumstances—of resorting to innumerable methods calculated to prevent childbirth, and, in many instances, to endanger their own lives—indicative of material advancement or barbarism? Do we live in an age of unparalleled mental and physical supremacy, or in one of moral and intellectual depravity? Are we a race of valetudinarians, incompetent to propagate our own species, or merely the degenerate offspring of an illustrious parentage?

To the casual observer such questions are, doubtless, of little moment; for probably few have paused to reflect on their immensity. To the physician, therefore, is assigned the rather unpleasant duty of discussing at length what must of necessity result from this aversion of many of our women to labor and its attending sufferings; and, while not unmindful of the delicacy which should clothe so portentious a subject, I, the least among you, and respecting that element of refinement, am constrained, nevertheless, to strip from it those vestments which, falsely worn, are emblematical of ignorance, stagnation, and skepticism.

Among our American women there is, apparently, no organized movement to induce an antagonism to procreation, but for some time the drift of society has been in this direction. The standard of social life is such that sexual promiscuity is tolerated as it has never been before; and, consequently, many of the elite—despite their opulence and adaptability—shirk marriage and the responsibilities imposed by its lofty and sublime creative work. However, it might be appropriately said, in justice to the opposite and weaker sex, that, as a rule, dissipation and degradation have invariably commenced with the men and been communicated by them to the women; but when they sink into that "slough of despond" —sexual lewdness and avoidance of maternity—the intelligent class must of necessity disappear, while civilization, robbed of the minds and morals which gave it birth, terminates its existence.

Reproduction may be defined as the production by living, organized bodies of others similar to themselves, and is essential for the maintenance of the race. Woman has been designated by nature as a temporary abiding place for the fertilization and development of the impregnated ovum. From her is derived its sustenance; and, conception having taken place, to her is delegated the faculty of protection during the period of gestation. Where man's obligation ends, woman's begins. To her, if he be a *man*, he looks for the life of another born in his own likeness. Upon her devolves, in many instances, the responsibility for this life. Too often is hers the power of ridding herself of herself. Feticide is as heinous as homicide. There can be no distinction nor difference. Legally, the expulsion of the contents of the uterus at any time determines an abortion; and the more recent laws of the United States and Great Britain fail to make any discrimination in the criminality of the act, whether before or after quickening. Whether the ovum shall pass through its various stages of evolution, and, at full term, issue forth from its uterine home a being perfectly and symmetrically formed, fashioned after the similitude of its progenitors, and destined to add new glory to maternity; or, to the contrary, meet an untimely and premeditated death with the sanction and assistance of one who had indulged in coition merely for the gratification of sensual lust and pleasure is, in this enlightened and progressive age, too frequently influenced by "wiseacres" who would make of conception a thing of ridicule and of motherhood a paradise for fools.

Nature is not frivolous. Her laws in regard to the relation and position of the sexes are not to be scornfully disregarded. Statistics prove that the married man has a better prospect of life than the celibate, and, all things being equal, the mother of a family than the spinster. Consequently, the prejudice of the laity against maternity on the ground that it endangers and shortens life—a doctrine making nature a fool, as one eminent writer declares—is a fitting testimonial to the ignorance and erroneous ideas of a pessimistic people.

Poverty has been frequently assigned as one of the many causes for the present unfortunate state of affairs, not a few advocating that strenuous circumstances demand that conception and birth be regulated and limited. A glance at labor statistics, however, will suffice to prove that the voluntary limitation of children always has been, in all civilizations and societies, by the intelligent and rich, and not by the poor and needy. As the editor of one of our Southern journals has rightly said: "The rich American man's wife scorns to be a mother. She leaves that responsibility to her French maid, her Irish seamstress, and her German washerwoman, and when their sons are noted, declares with contempt their humble origin." The province of Quebec is decidedly more prolific than New England despite climatic conditions, which have been advanced to account for the low birth rate, the weakness of reproduction among native Americans, and the replacement of the original American stock by immigrants. To quote from the very admirable work of a distinguished son of that soil: "Be it said to the detriment of higher education, the whole tendency of the New England method of bringing up young women is to cultivate ambitions inconsistent with maternity. The sexual instincts are effaced, and the God-like power of creation in motherhood is neglected, belittled, or condemned." Such a condition is not confined to this immediate section. Conceived in vice and nurtured in dishonor, its germs have increased and multiplied until now, apparently, the entire country is infected.

Even the women of our fair and sunny South, those whom we have delighted to praise in song and story, those whom we have cherished as personifications of virtue, purity, and right, have breathed into their characters and into the characters of their daughters these noxious organisms of infamy, ignominy, and disgrace. Maternity repudiated! That God-given privilege abhorred and basely scorned! Reproduction signifying nothing, and prattling infancy a loathsome canker, preying, like a vulture, on the flesh! Of a truth—

"It out-Herods Herod."

We have arrived at that point in our civilization where the cultivated avoid having children, or refuse to be bothered with them at all. In intelligent communities onanism, prevention of conception, abortion, or surgical interference is the rule; and marriage may be for companionship, for money, for lust—in fact, for any and everything save parturition. Note the families having less than three children, and in many instances none. Retrogression rather than progression is apparent. Whereas the average American family formerly numbered seven or ten, it is now almost an exception to find half that number. How many women have been taught to regard ignorance on sexual matters as a synonym of innocence, and glory in this lack of information! Unconscious of the "God-given possibilities involved in an educated maternity," they will resent any allusion made thereto by their family physician as "indelicate and indecent," without the remotest realization of its vital importance to them individually and collectively. Others will seek to substantiate their position by citing a medical writer who, in an article, asserted that "It is wiser to prevent pregnancy by one of the means mentioned [that is, through the instrumentality of the condom or pessary] than to allow it, and perhaps let the poor woman resort to means to destroy her offspring in other ways that are criminal, and that are much more destructive to their health." Can it be that our maternal ancestors were the antitheses of our modern society belles? Did an all-wise Creator see fit to construct their genital tracts on a different anatomical basis? Is the *fin de siècle* woman altogether sterile and, consequently, incapacitated for the lying-in period? Are we a psalm-singing nation of latter-day "saints" in whom the passion for sexual intercourse is eradicated, belittled, or condemned? You, gentlemen, who are accustomed to follow in the wake of gonorrhea, syphilis, and their delightful sequelæ could furnish some interesting testimony. How, then, can such a state be accounted for, save in the diabolical practices of the married people of to-day, and in the advice and instruction imparted to the coming generation, even in the bloom of youth? How many of this country's fairest daughters refrain from assuming the responsibilities attending a marriage state, or enter it with trepidation, because the mother of their childhood has instilled into their characters something of the lasciviousness and baseness of her own!

When pamphlets are distributed offering for sale books in which instruction may be found "how to produce abortion in a variety of ways, some of which are not known to the medical world"; and also "valuable hints as to the best means by which evasion of the law can be accomplished when a physician is so unfortunate as to be 'suspected' of having been guilty of such a step"; when we see in our daily papers that Dr. and Mrs. Blackleg "*positively guarantee* to immediately relieve the most obstinate cases of irregularities (from any cause) by the only absolutely *safe* and *painless* method of treatment," and that "every case is treated strictly upon *honor.*" When we read in conspicuous places on the pages of our leading publications that "we have unequalled accommodations for ladies who remain under treatment with the positive assurance of absolute *privacy,*" and that "an infallible cure is assured or no charge"; when the columns of the press are filled with vaunted nostrums to bring on the menses and notices of multitudinous types of "female regulators"; when the monthly magazines are replete with illustrations and literature on the "most perfect and reliable syringe obtainable," and the large retail drygoods houses publish in their annual catalogues matter of a similar kind and like rot too numerous to mention; when we note the many who grasp and hold tenaciously to the offers of these charlatans, and perceive in our acquaintance the few children to a marriage; when, cognizant of the number totally sterile, we ponder the decrease in the birth-rate from year to year, well may we conclude that American breeding is not, as a rule, done by its intelligent and dominant families. Well may we pause and ask of ourselves the question, What is our destiny?

In most European nations there is a steady decrease of the birth-rate. Statistics prove that the French are the least and the Germans the most prolific people—the number of children born alive annually per 1,000 women of 15 to 50 years, ranging from 102 with the former to 150 with the latter. When we consider the fact that of this number 80 children out of every 1,000 in Prussia and 84 in France are illegitimate, it is not difficult to appreciate that a healthy marriage adds little to the population. According to statistics recently published (*Medical Record*, April 17, 1897) the population of the former country in 1895 was less by nearly 17,000 than the year before; and this falling off, says that periodical, is "manifestly due to the decreasing birth-rate, which since 1890 has been below the death-rate." A comparison of the percentages by decades during the present century shows a progressive diminution. From 1801–10 there were 33 births per 1,000 inhabitants, and this rate has declined with each succeeding decade to 32, 31, 29, 27, 26, 25, and 24; and to 22 during the first half of the last (from 1891–95). Evidently the apprehension so widely felt that France, as a nation, is doomed to ultimate extinction, is justified. No accurate statistics for the United States exist; but the census of 1890 shows that we are following in the footsteps of our sister republic; and unless there can be, and is, originated a remedy, we will likewise progress to extermination. From 1870–80 inclusive, with an increase in population of 11,598,000 there was a corresponding increase from births of 8,891,000; whereas for the following decade with an increase in the former of 12,225,000 there is only an accession of 6,950,000 in the latter. In a population twenty-five per cent. larger in the last, there were consequently, 2,941,000 births less than in the first. Had the birth-rate been in the same proportion we should have had an increase of over 10,000,000 instead of the number actually recorded. Per 1,000 inhabitants the rate estimated by Dr. Billings, of the Census office, was, in 1890, about thirty-one. As to the falling off he says:—"It is probable that the most important factor in the change is the deliberate and voluntary avoidance or prevention of childbearing on the part of a steadily increasing number of married people, who not only prefer to have but few children, but know how to obtain their wish." Dr. Cyrus H. Edson voices these sentiments, but attributes the principal cause to a "physical and nervous deterioration of the women, largely due to the severe strain of modern life and education." The recent mayoralty report of one of our leading southern cities, with an approximate population of 56,000, shows the number of deaths for that year to have been between 1,700 and 1,800; or about 31 per 1,000 inhabitants. The births registered for the same period were 700 less, or 18 and a fraction for every 1,000 souls. Granting that there were many births unrecorded, and appreciating the difficulty in getting every physician in a community to promptly report the same to the health officer, the fact remains indisputable that the city under consideration has gained less than 2 per cent. in population during the last decade, from 1890 to 1900. While this may be attributable in a measure to the keen rivalry and commercial competition superinduced by the proximity of neighboring seaboard towns, yet the great discrepancy between deaths and births at least warrants the assumption that there is assignable a more potent factor, a more logical cause. The *Journal of the American Medical Association,* (Sept. 8, 1900), says:—"San José, Cal., had a death-rate of 12.04 per 1,000 for the year 1898–99 and of 12.12 per 1,000 for the years 1899-1900. The birth-rate for the same years was 9.06 and 9.64 per 1,000 respectively. For July of the same year, Los Angeles, in the same State, had 134 births and a mortality of 166. In 1899 there were 28,441 births in Philadelphia, a decrease of 649 over that of 1898, or a birth-rate of 1 to each 44 living persons." The death-rate was 1 to 52.23. These figures, purposely culled at random from statistics published in the leading medical periodicals, prove conclusively that no immediate section is at fault, but rather, that the infection is general. In substantiation thereof a prominent author, in speaking of the rarity of full-blooded Americans in New England, says : "In general conversation those of the old American stock seemed nearly unanimous in expressing disregard, distaste, or positive opposition to procreation. They made no bones about it. From the preacher to the prostitute, infants were intruders. Progeny was prevented, destroyed, occasionally endured and rarely sought or welcomed."

Far be it from me to deal in pessimism. The view, rather, of an optimist were more pleasing. Appreciating, however, the decrease in birth-rate from year to year and from decade to decade, in the face of such a condition, when our better class of women, apparently forgetting principle, ignoring honor and ridiculing the sanctity of the marriage bed, "convert themselves into a mere sewer" for the passage of the degraded passions and lusts of their libidinous husbands, what must be the conclusion? That as a nation we have already attained to the zenith of our glory ; that, without the inauguration of an early reformation, extermination and death must come ; that from a people of unprecedented enlightenment and courage, we will degenerate into a race of mental, moral and physical pygmies ; and that the present vital movement can only eventuate in the elimination of the old American stock through non-reproduction. This is manifest even at the present day in many places where the population is maintained by immigration or the descendants of aliens, and with them procreation is largely due "not to reason, but to useful superstition."

To summarize, in the forceful language of Mr. Kinney : "Intellectual inquiry invites infidelity. Skepticism has no soul, nor has it breeding power. Man must have a belief to be in earnest. The skeptics disappear. The superstitious survive, but progress cannot live without intellectual activity. This is incompatible with the infallibility demanded for the integrity of superstition. So long as there is progress there must be intellectual independence. Here then is the dilemma : Skepticism and Sterility—or Superstition and Stagnation. Progress to extermination, or perpetuation of life without improvement."

BIBLIOGRAPHY.

*Medical Record*, April 17th, 1897.
*Journal of the American Medical Association,* April 14th, 1900.
*Ibid,* August 14th, 1900.
*Ibid,* September 8th, 1900.
*Alkaloidal Clinic*, April, 1899.
*Reese: Medical Jurisprudence and Texicology.*
*Kinney: Conquest of Death.*
*Statistics: World's Almanac and Encyclopedia,* 1898.
*Ibid,* 1900.
*Georgia Journal of Medicine and Surgery,* August, 1900.

